# Genetic syndromes in paediatric alopecia areata: a systematic review

**DOI:** 10.1093/skinhd/vzaf080

**Published:** 2025-11-05

**Authors:** Megan Park, Emma Price, Cathryn Sibbald

**Affiliations:** Temerty Faculty of Medicine, University of Toronto, Toronto, ON, Canada; Temerty Faculty of Medicine, University of Toronto, Toronto, ON, Canada; Division of Dermatology, Department of Medicine, The Hospital for Sick Children, Toronto, ON, Canada

## Abstract

**Background:**

A wide variation of phenotypes is displayed by individuals with alopecia areata (AA), especially in the paediatric population.

**Objectives:**

To systematically search published studies to identify paediatric syndromes with AA and their clinical features, and to summarize the current state of their genetic elucidation.

**Methods:**

In accordance with the PRISMA guidelines, a systematic search of MEDLINE, Embase, CENTRAL and PubMed databases was performed. All original case reports, case series and observational studies describing AA in children (aged <18 years) with monogenic or chromosomal syndromes were included. Further searches in OMIM and Orphanet, and reviews, clinical guidelines and basic science studies were used to retrieve additional comprehensive information on each syndrome.

**Results:**

After title and abstract screening of 1426 studies, and full-text review of 224 studies, 64 met the inclusion criteria and are summarized in this review. Overall, the search identified 33 genetic syndromes with paediatric AA. Prevalence estimates were available for 79% (*n* = 26/33) of syndromes, with 45% (*n* = 15/33) of syndromes presenting in fewer than 1/1 000 000 individuals. Sixty-seven per cent (*n* = 22/33) of syndromes were fully genetically elucidated; 12% (*n* = 4/33) were partially elucidated; 9% (*n* = 3/33) were not genetically elucidated; and 12% (*n* = 4/33) were syndromes with chromosomal abnormalities. Seventy-nine per cent (*n* = 26/33) of syndromes were described by only one report, while 21% (*n* = 7/33) were described in multiple independent reports.

**Conclusions:**

Despite the limited knowledge of these syndromes, this review provides insights into the range of genetic syndromes with paediatric AA and their clinical features, facilitating early prediction, diagnosis and personalized treatments.

## Teaser

There is a wide variation of phenotypes displayed by individuals with alopecia areata (AA), especially in the paediatric population. This review aims to systematically search published studies to identify paediatric syndromes with AA and their clinical features, and to summarize the current state of their genetic elucidation. Overall, this review identifies and summarizes a total of 33 distinct syndromes associated with paediatric AA into a comprehensive catalogue.

What is already known about this topic?

- Alopecia areata (AA) is a common autoimmune form of nonscarring hair loss caused by T-cell-mediated damage to hair follicles.
- Genome-wide association studies facilitated by the National Alopecia Areata Registry have identified several genes linked to AA susceptibility, offering insights into its genetic basis.
- While 10–20% of patients report a family history, AA does not follow Mendelian inheritance and exhibits diverse phenotypes.

What does this study add?

- Overall, the search identified 33 genetic syndromes with paediatric AA.
- Prevalence estimates were available for 79% (*n* = 26/33) of syndromes, with 45% (*n* = 15/33) of syndromes presenting in fewer than 1/1 000 000 individuals; 67% (*n* = 22/33) of syndromes were fully genetically elucidated.
- This review offers a comprehensive catalogue, providing insights into the range of genetic syndromes, and facilitating early recognition and targeted investigations, as well as potentially personalized treatments.

Alopecia areata (AA) is a common form of nonscarring hair loss^1^ caused by T-cell-mediated damage to hair follicles.^2^ This autoimmune condition results in a variable pattern of relapsing or remitting hair loss in genetically susceptible individuals with unknown environmental triggers.^3^ Clinically, AA manifests in several patterns of hair loss: patchy AA, the most common pattern with small circular lesions usually on the scalp; alopecia totalis, complete loss of scalp hair; and alopecia universalis, complete loss of all body hair.^1^

The aetiology and pathogenesis of AA remain unclear. Historical hypotheses include infection; the trophoneurotic hypothesis, which suggests the association with AA onset and emotional or physical stress; thallium acetate poisoning; and thyroid disease and hormonal fluctuations.^1^ Recent transcriptional profiling of mouse and human AA skin revealed gene expression indicative of cytotoxic T-cell infiltration.^2^ Additionally, the National Alopecia Areata Registry facilitated genome-wide association studies from over 10 000 individuals, identifying multiple genes linked with AA susceptibility and permitting analysis of significant epidemiological and sociomedical issues.^4^ Concurrent rodent models also confirmed this association through quantitative trait locus mapping, a technique linking DNA variations to phenotype.^5^ Collectively, these studies characterize AA to be a complex, polygenic, immune-mediated disease.^1^

Research demonstrates that AA has a significant genetic component in many patients.^6^ It frequently occurs in individuals with similar genetic profiles, and 10–20% of patients report a family history of the condition.^7^ Strong associations exist between AA and genetic syndromes, including trisomy 21^8^ and autoimmune polyendocrinopathy syndrome type 1.^9^ The 29–37% prevalence of AA in individuals with autoimmune polyendocrinopathy syndrome type 1 suggests a role for genes on chromosome 21 and AA susceptibility.^6^ Further genetic studies have identified associations with major histocompatibility complex class I polypeptide-related sequence A polymorphisms^10^ and the HLA-D gene region with AA susceptibility,^11^ as well as polymorphisms in *MX1*, *AIRE* and *NOTCH4*.^6^

Despite these associations, AA does not follow a simple Mendelian inheritance pattern, displaying a broad range of phenotypes among affected individuals.^12^ More comprehensive studies are necessary to support the hypothesis that AA is a complex condition that can be influenced by multiple genes.^12^ The current literature lacks a summary of genetic syndromes presenting with AA. However, various case studies, case series and epidemiological studies highlight individuals with rare genetic syndromes and AA, suggesting that there are several diverse genetic syndromes associated with AA as a possible feature. A detailed review of these syndromes will assist scientists and clinicians to better understand AA, improving diagnosis, treatment and genetic counselling. This review aimed to systematically search the published literature to identify paediatric syndromes with AA and their clinical features, and to summarize the current understanding of their genetic bases.

## Materials and methods

This systematic review was conducted in adherence with the PRISMA guidelines^13^ (Table S1; see Supporting Information). The protocol for this study was registered with PROSPERO (CRD42024508219).

### Study inclusion criteria

Eligibility criteria for this review were established using the Population, Intervention, Comparator, Outcomes, and Study design (PICOS) framework. Studies were included if they met the predetermined criteria:

Population: any patient (aged <18 years) with a monogenic syndrome or chromosomal syndrome, with AA.

Intervention: if applicable, treatments for the genetic syndrome and/or AA.

Comparator: not required.

Outcomes: clinical or genetic features of a paediatric AA syndrome.

Study design: original articles with novel findings on a genetic syndrome with AA, such as cohort studies, case–control studies, cross-sectional studies, and case studies and/or case series. Studies with genetic syndromes caused by environmental factors or based on animal studies or cellular studies were excluded. Review articles and conference abstracts were excluded.

A comprehensive literature search of MEDLINE, Embase and PubMed was conducted on 31 January 2024. The search strategy included the keywords ‘alopecia areata’ AND ‘syndrome*’ to identify studies on the genetic syndromes with clinical features of AA (Table S2; see Supporting Information). The OMIM and Orphanet databases, which catalogue rare genetic disorders, were also manually searched with the same search terms to identify additional relevant syndromes.

Title, abstract and full-text screening was conducted independently by two reviewers (M.P. and E.P.) using Covidence (www.covidence.org). At the full-text screening stage, studies were excluded if they did not meet the PICOS eligibility criteria. Discrepancies were settled through consensus by both reviewers. There were no geographical or language restrictions.

### Data extraction and analysis

The following data were extracted from each included study: first author, year of study, study design, number of patients and their characteristics (age, ethnicity, sex), clinical features of the genetic syndrome, disease-causing gene, type(s) of mutation, pattern(s) of inheritance, status of the genetic elucidation and presentation of AA with corresponding treatments, if applicable. Furthermore, clinical heterogeneity and whether AA was a cardinal feature of the syndrome was recorded. The findings were tabulated into a summary table consisting of the final list of genetic syndromes; further data from OMIM and Orphanet, and reviews, clinical guidelines and basic science studies were used to retrieve additional, comprehensive information on each syndrome, such as prevalence or pattern of inheritance. Data extraction was collected independently by two reviewers (M.P. and E.P.). All conflicts were resolved by discussion between the two reviewers. The quality of each article was evaluated and assigned according to the Oxford Centre for Evidence-Based Medicine 2011 Levels of Evidence criteria.^14^

## Results

**Figure 1:**
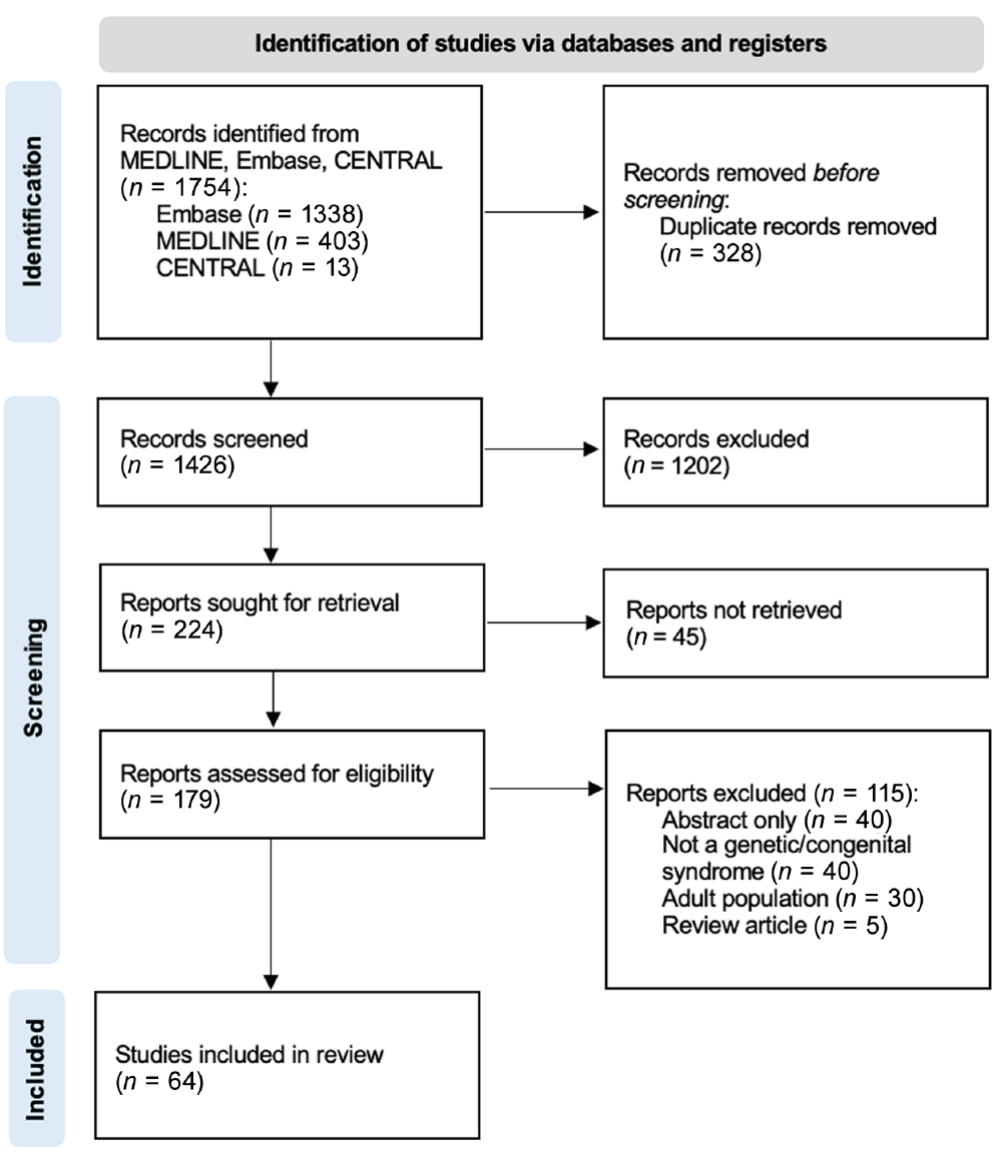
Preferred Reporting Items for Systematic reviews and Meta-Analyses (PRISMA) flow diagram for paediatric alopecia areata genetic syndromes. Edited from ref Page *et al*.^13^

After title and abstract screening of 1426 studies, and full-text review of 224 studies, 64 studies^15–78^ met the inclusion criteria and are summarized in this review (Figure 1). Original studies were reported from the USA (*n* = 8/64), China (*n* = 7/64), Turkey (*n* = 7/64), Italy (*n* = 5/64), Japan (*n* = 5/64), India (*n* = 5/64), Brazil (*n* = 4/64), Germany (*n* = 3/64), Saudi Arabia (*n* = 2/64), Singapore (*n* = 2/64), South Korea (*n* = 2/64), Israel (*n* = 2/64), Iran (*n* = 2/64), Canada (*n* = 1/64) Switzerland (*n* = 1/64), Ireland (*n* = 1/64), Greece (*n* = 1/64), Portugal (*n* = 1/64), the UK (*n* = 1/64), Romania (*n* = 1/64), Ukraine (*n* = 1/64), Poland (*n* = 1/64) and Spain (*n* = 1/64). The quality of each article was assessed: 75% (*n* = 48/64) were of Level 5 evidence consisting of case studies, 20% (*n* = 13/64) were Level 4 evidence consisting of case series and 5% (*n* = 3/64) were Level 2 evidence consisting of cross-sectional studies.

### Genetic syndromes

A total of 255 paediatric patients with a genetic syndrome and AA were included in this review. Among the 118 individuals who reported their sex, 65.3% (*n* = 77/118) were male and 34.7% (*n* = 41/118) were female. The mean (SD) reported age of patients was 10.9 (5.5) years. The search identified 33 genetic syndromes with AA. The genetic and clinical characteristics of all syndromes are summarized in Table 1 and Table S3 (see Supporting Information). The syndromes were organized into the following clinical presentations: dysmorphic features (*n* = 11/33), neurological features including developmental delays and seizures (*n* = 14/33), renal features (*n* = 2/33), hearing loss or auditory features (*n* = 3/33), ocular features (*n* = 12/33), cutaneous and nail features (*n* = 14/33), dental features (*n* = 3/33) and other (*n* = 3/33).

**Table 1:** Summary of genetic syndromes associated with alopecia areata in childhood

Genetic syndrome	No. of cases reported^a^ *n* (%)	Disease-causing gene(s) and type of inheritance	Dysmorphism	Neurological features	Renal anomalies	Hearing deficits or auditory features	Vision loss or ocular features	Dental anomalies	Other cutaneous	References^b^
Trisomy 21 (OMIM: #190675; ORPHA: 870)	148 (58.0)	21q22.3; sporadic	+	+	−	−	−	−	+	**^15–35^** ^,79^
Autoimmune polyglandular syndrome type 1 (OMIM: #240300; ORPHA: 3453)	10 (4.0)	*AIRE*; autosomal recessive	−	+	−	+	−	+	+	**^36–39^** ^,80,81^
Turner syndrome (OMIM: %300082; ORPHA: 881)	6 (2.4)	X monosomy; sporadic	+	+	−	−	−	−	+	**^40–45^** ^,82^
Incomplete antibody deficiency syndrome (ORPHA: 1006)	3 (1.2)	No data	−	+	−	−	−	−	+	**46** ^,83^
Knobloch syndrome (OMIM: #267750; ORPHA: 1571)	3 (1.2)	*COL18A1*; autosomal recessive	−	−	−	−	+	−	−	**^47^** ^,84^
Poretti–Boltshauser syndrome (OMIM: #615960; ORPHA: 370022)	3 (1.2)	*LAMA1*; autosomal recessive	−	+	−	−	+	−	−	**^47^** ^,85^
Autoimmune polyglandular syndrome type 3 (ORPHA: 227982)	2 (0.8)	No data; polygenic hereditary disease	−	−	−	−	−	−	−	**^48,49^** ^,86,87^
Chronic atypical neutrophilic dermatosis with lipodystrophy and elevated temperature (CANDLE) syndrome (OMIM #256040; ORPHA: 325004)	2 (0.8)	*PSMB8*; autosomal recessive	+	−	−	−	−	−	+	**^50,51^** ^,88^
Encephalocraniocutaneous lipomatosis (OMIM: #613001; ORPHA: 2396)	2 (0.8)	*FGFR1*; no data	−	+	−	−	+	−	−	**^52,53^** ^,89^
Immunodysregulation, polyendocrinopathy, enteropathy, X-linked (IPEX) syndrome (OMIM: #304790; ORPHA: 37042)	2 (0.8)	*FOXP3*; X-linked recessive	−	−	−	+	−	−	+	**^54,55^** ^,90,91^
Microcephaly-capillary malformation syndrome (OMIM: #614261; ORPHA: 294016)	2 (0.8)	*STAMBP*; autosomal recessive	+	+	−	+	+	−	+	**^56^,^92^** ^,93^
18p deletion syndrome (OMIM: #146390; ORPHA: 1598)	1 (0.4)	15.3-Mb deletion at 18p11.21p11.32; sporadic	+	+	−	−	−	−	−	**^57^** ^,94,95^
7p22.2 duplication syndrome	1 (0.4)	*SDK1*, *CARD11*; no data	+	+	−	−	−	−	−	**^58^** ^,96^
Acrocallosal syndrome (OMIM #200990; ORPHA: 36)	1 (0.4)	*KIF7*; autosomal recessive	+	+	−	−	−	−	−	**^59^** ^,97,98^
Adams–Oliver syndrome (OMIM #100300; ORPHA: 974)	1 (0.4)	*ARHGAP31*; autosomal dominant	−	+	−	−	−	−	−	**^60^** ^,99^
Aicardi–Goutières syndrome (OMIM #612952; ORPHA: 51)	1 (0.4)	*SAMHD1*; autosomal recessive	−	+	−	−	−	−	+	**^61^** ^,100^
Autoimmune polyglandular syndrome type 2 (OMIM %269200; ORPHA: 3143)	1 (0.4)	No data; polygenic hereditary disease	−	−	−	−	+	−	−	**^62^** ^,86^
Blepharophimosis–ptosis–epicanthus inversus syndrome (OMIM: #110100; ORPHA: 572333)	1 (0.4)	*FOXL2*; autosomal dominant, autosomal recessive	−	−	−	−	+	−	−	^**63**,101,102^
Bloom syndrome (OMIM: #210900; ORPHA: 125)	1 (0.4)	*RECQL3*; autosomal recessive	−	−	−	−	−	−	+	**^64^** ^,103,104^
Deficient in anterior pituitary function-variable immunodeficiency (DAVID) syndrome (OMIM: #615577; ORPHA: 293978)	1 (0.4)	*NFKB2*; autosomal dominant	−	−	−	−	−	−	−	**^65^** ^,105–107^
Oculo–auriculo–vertebral spectrum (OMIM: #164210; ORPHA: 141132)	1 (0.4)	*SF3B2*; autosomal dominant	+	−	−	−	−	−	−	**^66^** ^,108^
Gomez–Lopez–Hernandez syndrome (OMIM: %601853; ORPHA: 1532)	1 (0.4)	No data	+	−	−	−	+	−	−	**^67^** ^,109,110^
Hereditary hypotrichosis (Marie-Unna type) (OMIM: #146550; ORPHA: 444)	1 (0.4)	*U2HR*; autosomal dominant	−	−	−	−	+	−	−	**^68^** ^,111^
Louis-Bar syndrome (OMIM: #208900; ORPHA: 100)	1 (0.4)	*ATM*; autosomal recessive	−	−	−	−	−	−	−	**^69^** ^,112^
Kabuki syndrome (OMIM: #147920; ORPHA: 2322)	1 (0.4)	*KMT2D*; autosomal dominant	−	+	−	−	+	−	+	**^70^** ^,113,114^
Mayer–Rokitansky–Küster–Hauser syndrome (OMIM: %27700; ORPHA: 247775)	1 (0.4)	No data; autosomal dominant	−	−	+	−	−	−	−	**^71^** ^,115,116^
Metageria (premature ageing syndrome) (OMIM: 201200; ORPHA: 2600)	1 (0.4)	No data	−	−	−	−	+	−	+	**^72^** ^,117^
Oliver–McFarlane syndrome (OMIM: #275400; ORPHA: 3363)	1 (0.4)	*PNPLA6*; autosomal recessive	−	+	−	−	+	−	−	**^73^** ^,118,119^
Oro-facio-digital syndrome type 4 (OMIM: #258860; ORPHA: 2753)	1 (0.4)	*TCTN3*; autosomal recessive	+	+	−	−	+	+	−	**^74^** ^,120^ ^,121^
PLACK syndrome (OMIM: #616295; ORPHA: 444138)	1 (0.4)	*CAST*; autosomal recessive	−	−	−	−	−	−	+	**^75^** ^,122,123^
Ring chromosome 18 syndrome (OMIM: #601808; ORPHA: 1442)	1 (0.4)	Both ends of chromosome 18 are deleted and reunited to form a ring-shaped figure; autosomal dominant	+	−	−	−	−	−	+	**^76^** ^,124^
Van der Woude Syndrome 1 (OMIM: #119300; ORPHA: 888)	1 (0.4)	*IRF6*; autosomal dominant	−	−	−	−	−	−	+	**^77^** ^,125^
Wiedemann–Rautenstrauch syndrome (OMIM: #264090; ORPHA: 3455)	1 (0.4)	*POLR3A*; autosomal recessive	−	+	+	−	−	+	+	**^78^** ^,126^

^a^Total number of paediatric cases, *n* = 255.

^b^Bold references: original papers retrieved from the literature search; nonbold references: alternative sources.

All included syndromes demonstrate clinical heterogeneity. Sixty-seven per cent (*n* = 22/33) of syndromes were fully genetically elucidated; 12% (*n* = 4/33) were partially elucidated (7p22.2 duplication syndrome, autoimmune polyglandular syndrome type 2, autoimmune polyglandular syndrome type 3 and Mayer–Rokitansky–Küster–Hauser syndrome); 9% (*n* = 3/33) were not genetically elucidated (Gomez–Lopez–Hernandez syndrome, incomplete antibody deficiency syndrome and metageria); and 12% (*n* = 4/33) were syndromes with chromosomal abnormalities (trisomy 21, Turner syndrome, ring chromosome 18 syndrome and 18p deletion syndrome).

Prevalence estimates were available for 79% (*n* = 26/33) syndromes; of these 45% (*n* = 15/33) of syndromes presenting in fewer than 1/1 000 000 people. A total of 32/33 syndromes were described in the scientific literature or on databases (Orphanet and/or OMIM) using multiple names, while one was not assigned a name (‘7p22.2 duplication syndrome’). Seventy-nine per cent (*n* = 26/33) of syndromes were described by only one report, while 21% (*n* = 7/33) were described in multiple independent reports. The following syndromes with paediatric AA were described more than once in independent articles: autoimmune polyglandular syndrome type 1; autoimmune polyglandular syndrome type 3; chronic atypical neutrophilic dermatosis with lipodystrophy and elevated temperature (CANDLE) syndrome; trisomy 21; encephalocraniocutaneous lipomatosis; immune dysregulation, polyendocrinopathy, enteropathy, X-linked (IPEX) syndrome; and Turner syndrome.

Syndromes with alopecia as a cardinal feature had exceptionally low prevalence, estimated to be <1/1 000 000 for Gomez–Lopez–Hernandez syndrome, and there were no data on prevalence for hereditary hypotrichosis (Marie-Unna type) and incomplete antibody deficiency syndrome. The most common syndromes with regard to prevalence were autoimmune polyglandular syndrome type 2 (1/20 000), autoimmune polyglandular syndrome type 3 (1/20 000), Bloom syndrome (1/48 000 in Ashkenazi Jews), ­blepharophimosis-ptosis-epicanthus inversus syndrome (1/50 000) and Van der Woude syndrome 1 (1–9/100 000). Syndromes with chromosomal segmental abnormalities had the highest prevalence estimates, including 18p deletion syndrome (1/50 000), Turner syndrome (5/10 000) and trisomy 21 (1–5/10 000).

#### Alopecia areata clinical presentation

The onset of AA was described in 26.3% (*n* = 67/255) of individuals. Of these, the mean (SD) age at AA presentation was 6.8 (4.9) years. Meanwhile, the mean (SD) age of presentation of genetic syndrome was 1.9 (2.9) years (*n* = 78/255, 30.6%). Four per cent (*n* = 10/255) reported nail dystrophy along with AA. Only 4% (*n* = 9/255) of patients reported AA totalis or universalis. All patients with incomplete antibody syndrome (*n* = 3/9, 33%) reported AA totalis and universalis. One patient (*n* = 1/9, 11%) with autoimmune polyglandular syndrome type 3 had AA universalis with a family history of a grandfather with AA universalis, but no other comorbidities were reported.^48^ Two patients (*n* = 2/9, 22%) with trisomy 21 reported AA universalis, while three patients (*n* = 3/9, 33%) with trisomy 21 reported AA totalis. Treatments for AA were described in 9.4% (*n* = 24/255) of individuals: tacrolimus 0.1% ointment (*n* = 1/255); diphenylcyclopropenone 2% (*n* = 3/255); squaric acid dibutylester (*n* = 4/255); dinitrochlorobenzene 0.3% (*n* = 1/255); baricitinib (*n* = 1/255); clobetasol propionate 0.5 mg g^–1^ lotion and minoxidil 5% solution (*n* = 1/255); topical minoxidil 2% (*n* = 1/255); prednisone and tacrolimus (*n* = 1/255); tofacitinib (*n* = 2/255); and dithranol 0.2% with cysteine tablets and ciclosporin A (*n* = 9/255).

## Discussion

Previous studies have reported genes strongly associated with AA and other diseases; however, no reviews have specifically addressed associated genetic syndromes and paediatric AA. This systematic review identifies 33 genetic syndromes with paediatric AA, marking an important step for future research into the diagnosis, pathogenesis and treatment of syndromes presenting with AA. The literature now widely recognizes that AA represents a complex or multifactorial genetic trait.

Several syndromes identified in this review originate from specific geographical areas, potentially influencing the prevalence of inherited syndromes due to unique founder mutations arising from affected ancestors or consanguinity. The prevalence of children with AA and positive family history in the literature ranges from 8.4% to 51.6%.^127^ A review of the National AA Registry on childhood alopecia revealed that 25.4% children had a positive family history, with 8.1% having more than three affected first-degree relatives.^127^ One study from Kuwait reported that 51.6% of the cohort had a positive family history.^128^ The authors attributed this prevalence to the frequency of consanguineous marriages in their patient cohort, suggesting that a combination of environmental and genetic factors could precipitate AA.^129^

The syndromes identified in this review exhibit considerable genetic heterogeneity. Clinical heterogeneity within a syndrome may be due to genetic or allelic heterogeneity, ancestral differences and environmental impacts, including medical treatments and epigenetics. Gene–environment and gene–gene interactions also contribute to the heterogeneity. The type of mutations and structural variants such as insertions, deletions, inversions and complex rearrangements play a role, although their prevalence in these paediatric syndromes with AA is not well documented in the literature. Genome-wide association studies, linkage studies and whole-exome sequencing studies have identified links between AA and several genetic variants.^130,131^ These studies have identified functionally relevant disease genes in AA and proposes a mechanism of the genetic susceptibility in the disease. However, they characterize the role of the genetic variants on the population level and often lack specific patient data with AA as a feature, such as syndromic diagnoses. Our systematic review of 33 paediatric syndromes with AA reveals extensive phenotypic hetero­geneity; however, complete characterization of clinical heterogeneity is challenging due to its high prevalence in genetic syndromes and rarity of paediatric patients with syndromic AA.

Despite their rarity and heterogeneity, recognizing these syndromes can facilitate early identification, diagnosis and management of AA in affected children. Clinical and genetic overlap, along with the uncertain evolution of AA within the broader symptomatology of these syndromes, can complicate classification. In most case reports and other studies, it remains uncertain whether AA is a feature of the syndrome itself, related to a genetic defect, or whether it represents a coincidental comorbidity. Given the lack of detailed data on the onset and progression of symptoms in these conditions, genetic testing and molecular analyses may enhance diagnostic accuracy. Clinically, over half of these syndromes present with neurological deficits, particularly developmental delays, highlighting the need to investigate syndromic a etiologies in children with neurological features and AA. Similarly, nearly half exhibit dermatological manifestations, emphasizing the importance of comprehensive clinical evaluation to distinguish syndromic from nonsyndromic cases of AA. Challenging clinical presentations may warrant further investigation for underlying syndromic aetiologies of children with AA.

The studies identified in this review that report treatment of AA used standard AA treatments, in addition to therapies specific to the syndrome. Although resolution of AA following syndrome-specific treatment has not been reported, identifying genetic aetiologies of these syndromes allows for the establishment of novel molecular targets and personalized treatments. For example, *AIRE*, on chromosome 21, known for its role in autoimmune regulation, is strongly associated with AA.^132^ Additional studies are needed to explore personalized treatments for genetic syndromes and their impact on AA. One case in this review involved a boy with goitre, blepharoptosis and AA diagnosed with autoimmune polyglandular syndrome type 2 who was given methimazole for his hyperthyroidism.^62^ While methimazole can inhibit the immune–inflammatory response, it may induce alopecia.^133^ Managing drug interactions in paediatric syndromes with AA is crucial, as the genetic basis reviewed may facilitate novel therapeutic interventions. Advances have been made in genetic therapy, particularly using bone marrow-derived mononuclear cells and follicular stem cells as an effective treatment for AA.^134^ The identification of these paediatric syndromes with AA can be utilized in clinical and basic research studies, and support ongoing clinical trials.

This review has limitations. It predominantly relied on case reports and case series, constraining the depth of detail included for these syndromes. The limited documentation and low prevalence of these syndromes hinder comprehensive understanding and raise the possibility that the observed presentations with AA could be by chance alone. Additionally, the geographical variability of case reports limits the generalizability of prevalence data to the global population. Future research is needed to explore the autoimmune characteristics and genetic profiles of these syndromes, assess clinical heterogeneity and consolidate similar syndromes related to AA.

Despite these limitations, a major strength of our review is its comprehensive summary of genetic syndromes associated with paediatric AA. By utilizing multiple databases with additional searches of clinical guidelines, reviews and basic science literature, this review provides a thorough overview of the existing knowledge of these syndromes. Additionally, this review highlights the challenges related to identifying paediatric syndromes with AA, due to their low prevalence and variation in nomenclature.

Further clinical research and enhanced technological advancements in the field of genetics may elucidate these syndromes further and identify additional genes associated with paediatric AA. Our review provides novel insights into the diversity of genetic syndromes linked to paediatric AA. This comprehensive summary will assist clinicians in the early recognition and targeted investigations of patients with paediatric syndromes presenting with AA. We hope that this systematic review will contribute to further advancements in understanding the genetic pathophysiology of paediatric AA.

### Author contributions

Megan Park (Conceptualization [supporting], Data curation [lead], Formal analysis [lead], Investigation [lead], Methodology [lead], Project administration [equal], Writing—original draft [lead], Writing—review & editing [lead]), Emma Price (Data curation [lead], Investigation [supporting], Methodology [supporting], Writing—review & editing [supporting]), and Cathryn Sibbald (Conceptualization [lead], Methodology [supporting], Resources [lead], Supervision [lead], Writing—review & editing [lead])

## Supplementary Material

vzaf080_Supplementary_Data

## Data Availability

The data underlying this article are available in the article and in its online Supporting Information.
