# Supplementary material for: Genetic syndromes in paediatric alopecia areata: a systematic review
Source: Skin Health Dis. 2025 Nov 5;5(6):409–18. doi: 10.1093/skinhd/vzaf080 (PMC12648540; doi:10.1093/skinhd/vzaf080)
Supplement: vzaf080_Supplementary_Data [file vzaf080_supplementary_data.zip › AAGeneticSyndromes_Supplementary_Table_2.docx]

**Supplementary Table 2. Line-by-line search strategy**

| **Database:** Ovid MEDLINE: Epub Ahead of Print, In-Process & Other Non-Indexed Citations, Ovid MEDLINE® Daily and Ovid MEDLINE® <1946-Present> | | |
| --- | --- | --- |
| **#** | **Search Terms** | **Results** |
| 1 | exp Alopecia Areata/ | 4358 |
| 2 | alopecia areata.tw,kf. | 5489 |
| 3 | exp Syndrome/ | 123987 |
| 4 | syndrome*.tw,kf. | 1243729 |
| 5 | 1 or 2 | 6204 |
| 6 | 3 or 4 | 1265463 |
| **7** | **5 and 6** | **348** |
