# Supplementary material for: Genetic syndromes in paediatric alopecia areata: a systematic review
Source: Skin Health Dis. 2025 Nov 5;5(6):409–18. doi: 10.1093/skinhd/vzaf080 (PMC12648540; doi:10.1093/skinhd/vzaf080)
Supplement: vzaf080_Supplementary_Data [file vzaf080_supplementary_data.zip › SupplementaryTable3.docx]

**Supplementary Table 3**. Genetic syndromes with alopecia areata in childhood categorized by clinical features

| **Genetic Syndrome** | **Disease-causing gene(s) and type of inheritance** | **Total Sample Size** | **Prevalence** | **Sex (n), reported age (average, if applicable)** | **Clinical presentation of genetic syndrome (age of diagnosis or presentation, clinical features, etc.)** | **Clinical presentation of alopecia areata (age of presentation, location, treatment, etc.)** | **Other systems involved** | **References^a^** |
| --- | --- | --- | --- | --- | --- | --- | --- | --- |
| **Syndromes with dysmorphic features** | | | | | | | | |
| 18p Deletion Syndrome (OMIM: #146390; ORPHA:1598) | 15.3-Mb deletion at 18p11.21p11.32; sporadic | 1 | 1/50 000 | F, 7 | Diagnosed at age 7; short stature, scoliosis, genu varum, facial anomalies, intellectual disability, growth and developmental delay. | Scalp; no treatment reported. | Neurologic | **57,** 94, 95 |
| 7p22.2 Duplication Syndrome | *SDK1, CARD11*; no data | 1 | No data | M, 14 | Presented at age 1; developmental delay and dysmorphic features, toe-walking gait, malalignment of feet, proportionate short stature. | Left occipital region (2 x 3 cm); no treatment reported. | Neurologic | **58,** 96 |
| Acrocallosal syndrome (OMIM #200990; ORPHA:36) | *KIF7*; autosomal recessive | 1 | <1/1 000 000 | M, 4 | Presented at birth; cleft palate, bilateral inguinal hernia, cryptorchidism, bilateral polydactyly of the feet, severe developmental delay, severe hypotonia, brisk reflexes, macrocephaly, irregular cranial suture, frontal bossing, facial anomalies. | Frontal region; no treatment reported. | Neurologic | **59**, 97, 98 |
| Chronic atypical neutrophilic dermatosis with lipodystrophy and elevated temperature (CANDLE) syndrome (OMIM #256040; ORPHA:325004) | *PSMB8*; autosomal recessive | 2 | No data | M(2), 14.5 | Presented at 1 month with fever with urticarial and papular skin lesions; arthralgias, facial variations, eyelid swelling with a violaceous hue, bilateral mild hypertrichosis of his forehead, prominent lipodystrophy of cheeks with wrinkles around the mouth), swollen fingers, mild gynecomastia (patient 1); widespread annular violaceous skin lesions (patient 2). | Presented at age 10 with chronic patch-type AA on occipital scalp in ophiasis pattern (patient 2); past treatments include IV pulse methylprednisolone and oral methotrexate, oral prednisone, dithranol cream and minoxidil (no resolution); treated with baricitinib for 9 months (complete resolution). | Cutaneous | **50, 51,** 88 |
| Gomez-Lopez-Hernandez syndrome (OMIM: %601853; ORPHA:1532) | No data; autosomal recessive | 1 | <1/1 000 000 | M, 6 | Presented at age 1; craniosystosis, hypogonadism, growth delay, hypertelorism, low set ears, lack of innervation of right cornea. | Presented at birth; scalp; no treatment reported. | Ocular | **67,** 109, 110 |
| Microcephaly-capillary malformation syndrome (OMIM: #614261; ORPHA:294016) | *STAMBP*; autosomal recessive | 2 | <1/1 000 000 | M(2), 6.5 | Widespread macular lesions; abnormal facial features of hypertelorism, down slanting palpebral fissures; complex seizures; aspiration pneumonitis; lack of head control and visual tracking; peripheral and axial hypotonia and microcephaly; hypertelorism; large mouth, long philtrum, and large ears; small fingers and toenails; severe cognitive and motor delay; bi-frontal narrowing, hypertelorism, bilateral squint, high forehead, and epicanthal fold; bilateral moderate sensory hearing loss, abnormal MRI, nail dystrophy. | Patchy AA (patient 1); thick and dry scalp hair, premature greying and nail involvement (patient 2); no treatment reported. | Neurologic, auditory, ocular, cutaneous | **56,** 92, 93 |
| Oro-facio-digital syndrome type 4 (OMIM: #258860; ORPHA:2753) | *TCTN3*; autosomal recessive | 1 | <1/1 000 000 | M, 12 | Presented at birth; dysmorphism, delayed motor and developmental milestones, transverse slanting palpebral fissures, ptosis, hypertelorism and apparent microphthalmia of the left eye, abroad nasal root and hypoplastic alanasi, posteriorly rotated ears, high arched narrow palate, tongue nodules with a bifid tip, irregular hypertrophic gingiva and an alveolar ridge with dental caries, operated tongue frenula, hands and feet were short, hypospadius, right side optic nerve coloboma and left microphthalmia, cataract, and total retinal detachment. | Sparse hair on scalp and eyebrows; no treatment reported. | Nuerologic, ocular, dental | **74**, 120, 121 |
| Oculo-auriculo-vertebral spectrum (OMIM: #164210; ORPHA:141132) | *SF3B2*; autosomal dominant | 1 | 1-9/100 000 | F, 1 | Presented at birth; right ear and mouth abnormality, fusion abnormality in the partial vertebra at L1 and L2 levels. | Presented at birth; no treatment reported. |  | **66,** 108 |
| Ring chromosome 18 syndrome (OMIM: #601808; ORPHA:1442) | Both ends of chromosome 18 are deleted and reunited to form a ring-shaped figure; autosomal dominant | 1 | <1 / 1 000 000 | M, 14 | Presented at birth; flat nasal bridge, low set ears, nail dystrophy, and proximally placed thumbs. | Presented at age 10; temporal and occipital hairlines in a continuous band; bilateral eyebrows and eyelashes affected; present exclamation mark sign; nail dystrophy; treated with regional immunotherapy (no resolution); spontaneous remission reported a few months later | Cutaneous | **76,** 124 |
| Trisomy 21 (OMIM: #190675; ORPHA:870) | 21q22.3; sporadic | 148 | 1-5/10 000 | M(45)/F(23), 8.9 (n = 64) | Presented at birth, nail dystrophy. | Presented at average age 7.7; alopecia universalis (n = 2); alopecia totalis (n = 3); multiple lesions on scalp (n = 14); one lesion on scalp (n = 1); dystrophic nails (n = 1)^b^ | Neurologic, cutaneous | **15-35,** 79 |
| Turner syndrome (OMIM: %300082; ORPHA:881) | X monosomy (most common); sporadic | 6 | 5/10 000 live female births | F(6), 11 | Hypothyroidism, hypopituitarism,  short stature, halo nevi, wide chest, broadly spaced nipples, sexual infantilism, cognitive delay, anxiety, nail dystrophy, and depression. | Presented at age 6; scalp; nail dystrophy (n = 2)  Treated with topical corticosteroid creams and topical minoxidil 2%; DPCP for 3 months (partial response); topical steroids. | Neurologic, cutaneous | **40-45,** 82 |
| **Syndromes with neurological features** | | | | | | | | |
| 18p Deletion Syndrome (OMIM: #146390; ORPHA:1598) | 15.3-Mb deletion at 18p11.21p11.32; sporadic | 1 | 1/50 000 | F, 7 | Diagnosed at age 7; short stature, scoliosis, genu varum, facial anomalies, intellectual disability, growth and developmental delay. | Scalp; no treatment reported. | Dysmorphic | **57,** 94, 95 |
| 7p22.2 Duplication Syndrome | *SDK1, CARD11*; no data | 1 | No data | M, 14 | Presented at age 1; developmental delay and dysmorphic features, toe-walking gait, malalignment of feet, proportionate short stature. | Left occipital region (2 x 3 cm); no treatment reported. | Dysmorphic | **58,** 96 |
| Autoimmune polyglandular syndrome type 1 (OMIM: #240300; ORPHA:3453) | *AIRE*; autosomal recessive | 10 | 1-9/1 000 000 | M(3)/F(6), 8.6 | Presented at age 8 months-18 years; ectodermal manifestations (hypoplasia of teeth, nail dystrophy, tympanic membrane calcification, vitiligo), hypoparathyroidism, chronic mucocutaneous candidiasis, Addison's disease, adrenal insufficiency, diabetes mellitus, ovarian failure, seizures. | Presented at 9 months and 11 years (n = 2); scalp, eyebrows, eyelashes, nail dystrophy; treated with 2% diphenylcyclopropenone in acetone for 5 months (no response) (n = 1). | Auditory, cutaneous, dental | **36-39,** 80, 81 |
| Acrocallosal syndrome (OMIM #200990; ORPHA:36) | *KIF7*; autosomal recessive | 1 | <1/1 000 000 | M, 4 | Presented at birth; cleft palate, bilateral inguinal hernia, cryptorchidism, and bilateral polydactyly of the feet, severe developmental delay, severe hypotonia, and brisk reflexes, macrocephaly, irregular cranial suture, frontal bossing, facial anomalies. | Frontal region; no treatment reported. | Dysmorphic | **59**, 97, 98 |
| Adams-Oliver syndrome (OMIM #100300; ORPHA:974) | *ARHGAP31*; autosomal dominant | 1 | No data | M, 7 | Diagnosed at age 7; complex cerebral arteriovenous thrombus leading to a stroke, new onset double diabetes complicated by diabetic ketoacidosis and hyperosmolar hyperglycaemic state. | Scalp; no treatment reported. |  | **60,** 99 |
| Aicardi-Goutieres syndrome (OMIM #612952; ORPHA:51) | *SAMHD1*; autosomal recessive | 1 | No data | F, 15 | Since birth; neonatal-onset encephalopathy, severe global developmental delay, bilateral knee contractures, vitiligo, microcephaly, spastic quadriparesis, non-verbal. | Presented at age 14; scalp; no treatment reported. | Cutaneous | **61,** 100 |
| Encephalocraniocutaneous lipomatosis (OMIM: #613001; ORPHA:2396) | *FGFR1*; no data | 2 | <1/1 000 000 | M(1)/F(1), 5 | Presented at 2 months with colobomas in the left eye, strabismus, vomiting, headaches, and seizures (patient 1); presented at age 3 with refractory uncontrolled seizures, mild developmental delay, right anophthalmia with an orbital calcified mass, lipomatosis in the right temporal area (patient 2). | Presented at 2 months; occipital region; no treatment reported. | Ocular | **52, 53,** 89 |
| Incomplete antibody deficiency syndrome (ORPHA:1006) | No data | 3 | No data | M(2)/F(1), 12 | Recurrent pyogenic infections of the skin and respiratory tract; delay in speech; small tonsils; one patient treated with gamma globulin therapy. | Presented at average age 7.3; all patients had AA universalis/totalis; no treatment reported. | Cutaneous | **46,** 83 |
| Kabuki syndrome (OMIM: #147920; ORPHA:2322) | *KMT2D*; autosomal dominant | 1 | 1-9 / 100 000 | M, 5 | Diagnosed at age 5; hypoglycemia, seizures, urinary system anomalies, severe mental retardation, recurrent pneumonia, recurrent otitis media, blue sclerae, prominent finger pads, joint hypermobility, cleft or high-arched palate, undescended testis, ptosis, long, curved eyelashes, ectodermal abnormalities, congenital heart defect, suborbital puffiness, atrial septal defect. | No description reported. | Ocular, cutaneous | **70,** 113, 114 |
| Microcephaly-capillary malformation syndrome (OMIM: #614261; ORPHA:294016) | *STAMBP*; autosomal recessive | 2 | <1/1 000 000 | M(2), 6.5 | Widespread macular lesions; abnormal facial features of hypertelorism, down slanting palpebral fissures; complex seizures; aspiration pneumonitis; lack of head control and visual tracking; peripheral and axial hypotonia and microcephaly; hypertelorism; large mouth, long philtrum, and large ears; small fingers and toenails; severe cognitive and motor delay; bi-frontal narrowing, hypertelorism, bilateral squint, high forehead, and epicanthal fold; bilateral moderate sensory hearing loss, abnormal MRI, nail dystrophy. | Patchy AA (patient 1); thick and dry scalp hair, premature greying and nail involvement (patient 2); no treatment reported. | Dysmorphic, auditory, ocular, cutaneous | **56,** 92, 93 |
| Oliver-McFarlane syndrome (OMIM: #275400; ORPHA:3363) | *PNPLA6*; autosomal recessive | 1 | <1/1 000 000 | M, 5 | Presented at age 5 with trichomegaly, gross motor and language delays, slight growth delay, and bilateral chorioretinal degeneration. | Scalp; no treatment reported. | Ocular | **73**, 118, 119 |
| Oro-facio-digital syndrome type 4 (OMIM: #258860; ORPHA:2753) | TCTN3 gene; autosomal recessive | 1 | <1/1 000 000 | M, 12 | Presented at birth; dysmorphism, delayed motor and developmental milestones, transverse slanting palpebral fissures, ptosis, hypertelorism and apparent microphthalmia of the left eye, abroad nasal root and hypoplastic alanasi, posteriorly rotated ears, high arched narrow palate, tongue nodules with a bifid tip, irregular hypertrophic gingiva and an alveolar ridge with dental caries, operated tongue frenula, hands and feet were short, hypospadius, right side optic nerve coloboma and left microphthalmia, cataract, and total retinal detachment. | Sparse hair on scalp and eyebrows; no treatment reported. | Dysmorphic, ocular, dental | **74**, 120, 121 |
| Poretti-Boltshauser syndrome (OMIM: #615960; ORPHA:370022) | *LAMA1*; autosomal recessive | 3 | <1 /1 000 000 | M(2)/F(1),  2 | Diagnosed at average age 2, all patients presented with early onset myopia, two patients with cerebellar dysplasia, and one patient with parietal skull defect. | No description reported. | Ocular | **47**, 85 |
| Trisomy 21 (OMIM: #190675; ORPHA:870) | 21q22.3; sporadic | 148 | 1-5/10 000 | M(45)/F(23), 8.9 (n = 64) | Presented at birth, developmental delays, nail dystrophy. | Presented at average age 7.7; alopecia universalis (n = 2); alopecia totalis (n = 3); multiple lesions on scalp (n = 14); one lesion on scalp (n = 1); dystrophic nails (n = 1) | Dysmorphic, cutaneous | **15-35,** 79 |
| Turner syndrome (OMIM: %300082; ORPHA:881) | X monosomy (most common); sporadic | 6 | 5/10 000 live female births | F(6), 11 | Hypothyroidism, hypopituitarism,  short stature, halo nevi, wide chest, broadly spaced nipples, sexual infantilism, cognitive delay, anxiety, nail dystrophy, and depression. | Presented at age 6; scalp; nail dystrophy (n = 2)  Treated with topical corticosteroid creams and topical minoxidil 2%; DPCP for 3 months (partial response); topical steroids. | Dysmorphic, cutaneous | **40-45,** 82 |
| Wiedemann-Rautenstrauch syndrome (OMIM: #264090; ORPHA:3455) | *POLR3A*; autosomal recessive | 1 | <1 /1 000 000 | F, 5 | Presented at birth; left upper eyelid entropion, asymmetric septal hypertrophy and patent foramen ovale, nephrolithiasis, kidney stones, local lopoatrophy, osteopenia, natal teeth, delayed dentition, developmental delay. | No description reported. | Ocular, renal, dental | **78,** 126 |
| **Syndromes with renal features** | | | | | | | | |
| Mayer‑Rokitansky‑Küster‑Hauser Syndrome (OMIM: %27700; ORPHA:247775) | No data; autosomal dominant | 1 | 1-9 / 100 000 | F, 17 | Presented at age 17; right renal agenesis, hypoplastic vagina, primary amenorrhea, normal secondary sex characteristics. | Presented at age 17; ophiasis pattern; lymphocytic infiltration around the lower third of the hair follicle; no treatment reported. |  | **71,** 115, 116 |
| Wiedemann-Rautenstrauch syndrome (OMIM: #264090; ORPHA:3455) | *POLR3A*; autosomal recessive | 1 | <1 /1 000 000 | F, 5 | Presented at birth; left upper eyelid entropion, asymmetric septal hypertrophy and patent foramen ovale, nephrolithiasis, kidney stones, local lipoatrophy, osteopenia, natal teeth, delayed dentition, developmental delay. | No description reported. | Neurologic, ocular, dental | **78,** 126 |
| **Syndromes with hearing loss or auditory features** | | | | | | | | |
| Autoimmune polyglandular syndrome type 1 (OMIM: #240300; ORPHA:3453) | *AIRE*; autosomal recessive | 10 | 1-9/1 000 000 | M(3)/F(6), 8.6 | Presented at age 8 months-18 years; ectodermal manifestations (hypoplasia of teeth, nail dystrophy, tympanic membrane calcification, vitiligo), hypoparathyroidism, chronic mucocutaneous candidiasis, Addison's disease, adrenal insufficiency, diabetes mellitus, ovarian failure, seizures. | Presented at 9 months and 11 years (n = 2); scalp, eyebrows, eyelashes, nail dystrophy; treated with 2% diphenylcyclopropenone in acetone for 5 months (n = 1) (no response). | Neurologic, cutaneous, dental | **36-39,** 80, 81 |
| Immunodysregulation, polyendocrinopathy, and enteropathy, X-linked (IPEX) syndrome (OMIM: #304790; ORPHA:37042) | *FOXP3*; X-linked recessive | 2 | No data | M(2), 13.5 | Diagnosed at average age 13.5; eczema, food allergy, rhinitis, asthma, selective IgA deficiency, Idiopathic Juvenile Arthritis, IBD, vitiligo; autoimmune hemolysis, chronic otitis media and bronchiectasis, diarrhea alternative constipation, anemic appearance, purulence bilateral external auditory canal, and hearing loss. | Presented at age 12; scalp; treated with prednisone and tacrolimus (complete resolution). | Cutaneous | **54, 55,** 90, 91 |
| Microcephaly-capillary malformation syndrome (OMIM: #614261; ORPHA:294016) | *STAMBP*; autosomal recessive | 2 | <1/1 000 000 | M(2), 6.5 | Widespread macular lesions; abnormal facial features of hypertelorism, down slanting palpebral fissures; complex seizures; aspiration pneumonitis; lack of head control and visual tracking; peripheral and axial hypotonia and microcephaly; hypertelorism; large mouth, long philtrum, and large ears; small fingers and toenails; severe cognitive and motor delay; bi-frontal narrowing, hypertelorism, bilateral squint, high forehead, and epicanthal fold; bilateral moderate sensory hearing loss, abnormal MRI, nail dystrophy. | Patchy AA (patient 1); thick and dry scalp hair, premature greying and nail involvement (patient 2); no treatment reported. | Dysmorphic, neurologic, ocular, cutaneous | **56,** 92, 93 |
| **Syndromes with ocular features** | | | | | | | | |
| Autoimmune polyglandular syndrome type 2 (OMIM %269200; ORPHA:3143) | No data; polygenic hereditary disease | 1 | 1/20 000 | M, 9 | Presented at age 9; 7 years of goitre, 4 years of blepharoptosis; Hashimoto’s thyroiditis and myasthenia gravis; treated with methimazole, pyridostigmine bromide, and local injection of dexamethasone to thyroid. | Presented at 9 years; scalp; no treatment reported. |  | **62**, 86 |
| Blepharophimosis-Ptosis-Epicanthus Inversus Syndrome (OMIM: #110100; ORPHA:572333) | *FOXL2*; autosomal dominant, autosomal recessive | 1 | 1/50 000 | F, 6 | Since birth; third-degree consanguineous marriage; eyelid defects (abnormally wide eyes with narrow palpebral fissures and drooping eyelids), narrow palpebral fissures, bilaterally symmetrical ptosis and telecanthus. | Presented at 18 months; patchy hair loss on large areas of scalp and body hair; treated with topical immunotherapy with diphenylcyclopropenone for 10 months (complete resolution) |  | **63,** 101, 102 |
| Encephalocraniocutaneous lipomatosis (OMIM: #613001; ORPHA:2396) | *FGFR1*; no data | 2 | <1/1 000 000 | M(1)/F(1), 5 | Presented at 2 months with colobomas in the left eye, strabismus, vomiting, headaches, and seizures (patient 1); presented at age 3 with refractory uncontrolled seizures, mild developmental delay, right anophthalmia with an orbital calcified mass, lipomatosis in the right temporal area (patient 2). | Presented at 2 months; occipital region; no treatment reported. | Neurologic | **52, 53,** 89 |
| Gomez-Lopez-Hernandez syndrome (OMIM: %601853; ORPHA:1532) | No data | 1 | <1/1 000 000 | M, 6 | Presented at age 1; craniosystosis, hypogonadism, growth delay, hypertelorism, low set ears, lack of innervation of right cornea. | Presented at birth; scalp; no treatment reported. | Dysmorphic | **67,** 109, 110 |
| Kabuki syndrome (OMIM: #147920; ORPHA:2322) | *KMT2D*; autosomal dominant | 1 | 1-9 / 100 000 | M, 5 | Diagnosed at age 5; hypoglycemia, seizures, urinary system anomalies, severe mental retardation, recurrent pneumonia, recurrent otitis media, blue sclerae, prominent finger pads, joint hypermobility, cleft or high-arched palate, undescended testis, ptosis, long, curved eyelashes, ectodermal abnormalities, congenital heart defect, suborbital puffiness, atrial septal defect, typical facial features | No description reported. | Neurologic, cutaneous | **70,** 113, 114 |
| Knobloch syndrome (OMIM: #267750; ORPHA:1571) | *COL18A1*; autosomal recessive | 3 | <1/1 000 000 | M(3), 10 | Diagnosed at average age 10; all patients presented with early onset myopia; one patient had occipital skull defect. | No description reported. |  | **47,** 84 |
| Hereditary Hypotrichosis (Marie-Unna type) (OMIM: #146550; ORPHA:444) | *U2HR*; autosomal dominant | 1 | No data | F, 8 | Presented at age 8; gradual deterioration of central vision, diagnosed with Stargardt maculopathy. | Occipital and temporal areas with coarse, sparsely distributed hair on scalp; no treatment reported. |  | **68,** 111 |
| Metageria (premature aging syndrome) (OMIM: 201200; ORPHA:2600) | No data | 1 | No data | F, 8 | Tall and thin stature, facial atrophy, telangiectasias, prominent eyes, and loss of subcutaneous fat on limbs. | Presented at birth; scalp and eyebrows; no treatment reported. | Cutaneous | **72**, 117 |
| Microcephaly-capillary malformation syndrome (OMIM: #614261; ORPHA:294016) | *STAMBP*; autosomal recessive | 2 | <1/1 000 000 | M(2), 6.5 | Widespread macular lesions; abnormal facial features of hypertelorism, down slanting palpebral fissures; complex seizures; aspiration pneumonitis; lack of head control and visual tracking; peripheral and axial hypotonia and microcephaly; hypertelorism; large mouth, long philtrum, and large ears; small fingers and toenails; severe cognitive and motor delay; bi-frontal narrowing, hypertelorism, bilateral squint, high forehead, and epicanthal fold; bilateral moderate sensory hearing loss, abnormal MRI, nail dystrophy. | Patchy AA (patient 1); thick and dry scalp hair, premature greying and nail involvement (patient 2); no treatment reported. | Dysmorphic, neurologic, auditory, cutaneous | **56,** 92, 93 |
| Oliver-McFarlane syndrome (OMIM: #275400; ORPHA:3363) | *PNPLA6*; autosomal recessive | 1 | <1/1 000 000 | M, 5 | Presented at age 5 with trichomegaly, gross motor and language delays, slight growth delay, and bilateral chorioretinal degeneration. | Scalp; no treatment reported. | Neurologic | **73**, 118, 119 |
| Oro-facio-digital syndrome type 4 (OMIM: #258860; ORPHA:2753) | *TCTN3*; autosomal recessive | 1 | <1/1 000 000 | M, 12 | Presented at birth; dysmorphism, delayed motor and developmental milestones, transverse slanting palpebral fissures, ptosis, hypertelorism and apparent microphthalmia of the left eye, abroad nasal root and hypoplastic alanasi, posteriorly rotated ears, high arched narrow palate, tongue nodules with a bifid tip, irregular hypertrophic gingiva and an alveolar ridge with dental caries, operated tongue frenula, hands and feet were short, hypospadius, right side optic nerve coloboma and left microphthalmia, cataract, and total retinal detachment. | Sparse hair on scalp and eyebrows; no treatment reported. | Dysmorphic, neurologic, dental | **74**, 120, 121 |
| Poretti-Boltshauser syndrome (OMIM: #615960; ORPHA:370022) | *LAMA1*; autosomal recessive | 3 | <1 /1 000 000 | M(2)/F(1),  2 | Diagnosed at average age 2, all patients presented with early onset myopia, two patients with cerebellar dysplasia, and one patient with parietal skull defect. | No description reported. | Neurologic | **47,** 85 |
| Wiedemann-Rautenstrauch syndrome (OMIM: #264090; ORPHA:3455) | *POLR3A*; autosomal recessive | 1 | <1 /1 000 000 | F, 5 | Presented at birth; left upper eyelid entropion, asymmetric septal hypertrophy and patent foramen ovale, nephrolithiasis, kidney stones, local lipoatrophy, osteopenia, natal teeth, delayed dentition, developmental delay. | No description reported. | Neurologic, renal, dental | **78,** 126 |
| **Syndromes with other cutaneous and nail features** | | | | | | | | |
| Aicardi-Goutieres syndrome (OMIM #612952; ORPHA:51) | *SAMHD1*; autosomal recessive | 1 | No data | F, 15 | Since birth; neonatal-onset encephalopathy, severe global developmental delay, bilateral knee contractures, vitiligo, microcephaly, spastic quadriparesis, non-verbal. | Presented at age 14; scalp; no treatment reported. | Neurologic | **61,** 100 |
| Autoimmune polyglandular syndrome type 1 (OMIM: #240300; ORPHA:3453) | *AIRE*; autosomal recessive | 10 | 1-9/1 000 000 | M(3)/F(6), 8.6 | Presented at age 8 months-18 years; ectodermal manifestations (hypoplasia of teeth, nail dystrophy, tympanic membrane calcification, vitiligo), hypoparathyroidism, chronic mucocutaneous candidiasis, Addison's disease, adrenal insufficiency, diabetes mellitus, ovarian failure, seizures. | Presented at 9 months and 11 years (n = 2); scalp, eyebrows, eyelashes, nail dystrophy; treated with 2% diphenylcyclopropenone in acetone for 5 months (n = 1) (no response). | Neurologic, auditory, dental | **36-39,** 80, 81 |
| Bloom Syndrome (OMIM: #210900; ORPHA:125) | *RECQL3*; autosomal recessive | 1 | 1/48 000 in Ashkenazi Jew | F, 12 | Presented at 3 months; erythematous eruption with blisters on face, exacerbating after exposure to sunlight, resembling lupus erythematosus; flat nose, reticular pigmentation on the forehead and trunk, finger swelling; shortened distal phalanges, wide fingernails. | No description reported. |  | **64,** 103, 104 |
| Chronic atypical neutrophilic dermatosis with lipodystrophy and elevated temperature (CANDLE) syndrome (OMIM #256040; ORPHA:325004) | *PSMB8*; autosomal recessive | 2 | No data | M(2), 14.5 | Presented at 1 month with fever with urticarial and papular skin lesions; arthralgias, facial variations, eyelid swelling with a violaceous hue, bilateral mild hypertrichosis of his forehead, prominent lipodystrophy of cheeks with wrinkles around the mouth), swollen fingers, mild gynecomastia (patient 1); widespread annular violaceous skin lesions (patient 2). | Presented at age 10 with chronic patch-type AA on occipital scalp in ophiasis pattern (patient 2); past treatments include IV pulse methylprednisolone and oral methotrexate, oral prednisone, dithranol cream and minoxidil (no resolution); treated with baricitinib for 9 months (complete resolution). | Dysmorphic | **50, 51,** 88 |
| Immunodysregulation, polyendocrinopathy, and enteropathy, X-linked (IPEX) syndrome (OMIM: #304790; ORPHA:37042) | *FOXP3*; X-linked recessive | 2 | No data | M(2), 13.5 | Diagnosed at average age 13.5; eczema, food allergy, rhinitis, asthma, selective IgA deficiency, Idiopathic Juvenile Arthritis, IBD, vitiligo; autoimmune hemolysis, chronic otitis media and bronchiectasis, diarrhea alternative constipation, anemic appearance, purulence bilateral external auditory canal, and hearing loss. | Presented at age 12; scalp; treated with prednisone and tacrolimus (complete resolution). | Auditory | **54, 55,** 90, 91 |
| Incomplete antibody deficiency syndrome (ORPHA:1006) | No data | 3 | No data | M(2)/F(1), 12 | Recurrent pyogenic infections of the skin and respiratory tract; delay in speech; small tonsils; one patient treated with gamma globulin therapy. | Presented at average age 7.3; all patients had AA universalis/totalis; no treatment reported. | Neurologic | **46,** 83 |
| Kabuki syndrome (OMIM: #147920; ORPHA:2322) | *KMT2D*; autosomal dominant | 1 | 1-9 / 100 000 | M, 5 | Diagnosed at age 5; hypoglycemia, seizures, urinary system anomalies, severe mental retardation, recurrent pneumonia, recurrent otitis media, blue sclerae, prominent finger pads, joint hypermobility, cleft or high-arched palate, undescended testis, ptosis, long, curved eyelashes, ectodermal abnormalities, congenital heart defect, suborbital puffiness, atrial septal defect, typical facial features | No description reported. | Neurologic, ocular | **70,** 113, 114 |
| Microcephaly-capillary malformation syndrome (OMIM: #614261; ORPHA:294016) | *STAMBP*; autosomal recessive | 2 | <1/1 000 000 | M(2), 6.5 | Widespread macular lesions; abnormal facial features of hypertelorism, down slanting palpebral fissures; complex seizures; aspiration pneumonitis; lack of head control and visual tracking; peripheral and axial hypotonia and microcephaly; hypertelorism; large mouth, long philtrum, and large ears; small fingers and toenails; severe cognitive and motor delay; bi-frontal narrowing, hypertelorism, bilateral squint, high forehead, and epicanthal fold; bilateral moderate sensory hearing loss, abnormal MRI, nail dystrophy. | Patchy AA (patient 1); thick and dry scalp hair, premature greying and nail involvement (patient 2); no treatment reported. | Dysmorphic, neurologic, auditory, ocular | **56,** 92, 93 |
| Metageria (premature aging syndrome) (OMIM: 201200; ORPHA:2600) | No data | 1 | No data | F, 8 | Tall and thin stature, facial atrophy, telangiectasias, prominent eyes, and loss of subcutaneous fat on limbs. | Presented at birth; scalp and eyebrows; no treatment reported. | Ocular | **72**, 117 |
| PLACK syndrome (OMIM: #616295; ORPHA:444138) | *CAST*; autosomal recessive | 1 | <1/1 000 000 | M, 11 | Presented at 3 months; bullae, skin peeling, generalized xerosis, punctate palmoplantar keratoderma, nail changes (proximal leukonychia, distal onycholysis, partial nail dystrophy). | Scalp; nail dystrophy; no treatment reported |  | **75,** 122, 123 |
| Ring chromosome 18 syndrome (OMIM: #601808; ORPHA:1442) | Both ends of chromosome 18 are deleted and reunited to form a ring-shaped figure; autosomal dominant | 1 | <1 / 1 000 000 | M, 14 | Presented at birth; flat nasal bridge, low set ears, nail dystrophy, and proximally placed thumbs. | Presented at age 10; temporal and occipital hairlines in a continuous band; bilateral eyebrows and eyelashes affected; present exclamation mark sign; nail dystrophy; treated with regional immunotherapy (no resolution); spontaneous remission reported a few months later | Dysmorphic | **76,** 124 |
| Trisomy 21 (OMIM: #190675; ORPHA:870) | 21q22.3; sporadic | 148 | 1-5/10 000 | M(45)/F(23), 8.9 (n = 64) | Presented at birth, nail dystrophy. | Presented at average age 7.7; alopecia universalis (n = 2); alopecia totalis (n = 3); multiple lesions on scalp (n = 14); one lesion on scalp (n = 1); dystrophic nails (n = 1) | Dysmorphic, neurologic | **15-35,** 79 |
| Turner syndrome (OMIM: %300082; ORPHA:881) | X monosomy; sporadic | 6 | 5/10 000 live female births | F(6), 11 | Hypothyroidism, hypopituitarism,  short stature, halo nevi, wide chest, broadly spaced nipples, sexual infantilism, cognitive delay, anxiety, nail dystrophy, and depression. | Presented at age 6; scalp; nail dystrophy (n = 2)  Treated with topical corticosteroid creams and topical minoxidil 2%; DPCP for 3 months (partial response); topical steroids. | Neurologic, dysmorphic | **40-45,** 82 |
| Van der Woude Syndrome 1 (OMIM: #119300; ORPHA:888) | *IRF6*; autosomal dominant | 1 | 1-9 / 100 000 | M, 9 | Presented at birth; cleft palate, median lower lip pit, fingernail pitting and trachyonychia on all digits. | Presented at age 9; 70% of scalp and eyebrows affected; fingernail pitting and trachyonychia on all digits, with normal toenails; treated with clobetasol 0.5 mg/g lotion and minoxidil 5% solution (partial resolution). |  | **77,** 125 |
| **Syndromes with dental findings** | | | | | | | | |
| Autoimmune polyglandular syndrome type 1 (OMIM: #240300; ORPHA:3453) | *AIRE*; autosomal recessive | 10 | 1-9/1 000 000 | M(3)/F(6), 8.6 | Presented at age 8 months-18 years; ectodermal manifestations (hypoplasia of teeth, nail dystrophy, tympanic membrane calcification, vitiligo), hypoparathyroidism, chronic mucocutaneous candidiasis, Addison's disease, adrenal insufficiency, diabetes mellitus, ovarian failure, seizures. | Presented at 9 months and 11 years (n = 2); scalp, eyebrows, eyelashes, nail dystrophy; treated with 2% diphenylcyclopropenone in acetone for 5 months (n = 1) (no response). | Neurologic, auditory, cutaneous | **36-39,** 80, 81 |
| Oro-facio-digital syndrome type 4 (OMIM: #258860; ORPHA:2753) | *TCTN3*; autosomal recessive | 1 | <1/1 000 000 | M, 12 | Presented at birth; dysmorphism, delayed motor and developmental milestones, transverse slanting palpebral fissures, ptosis, hypertelorism and apparent microphthalmia of the left eye, abroad nasal root and hypoplastic alanasi, posteriorly rotated ears, high arched narrow palate, tongue nodules with a bifid tip, irregular hypertrophic gingiva and an alveolar ridge with dental caries, operated tongue frenula, hands and feet were short, hypospadias, right side optic nerve coloboma and left microphthalmia, cataract, and total retinal detachment. | Sparse hair on scalp and eyebrows; no treatment reported. | Dysmorphic, neurologic, ocular | **74**, 120, 121 |
| Wiedemann-Rautenstrauch syndrome (OMIM: #264090; ORPHA:3455) | *POLR3A*; autosomal recessive | 1 | <1 /1 000 000 | F, 5 | Presented at birth; left upper eyelid entropion, asymmetric septal hypertrophy and patent foramen ovale, nephrolithiasis, kidney stones, local lipoatrophy, osteopenia, natal teeth, delayed dentition, developmental delay. | No description reported. | Neurologic, renal, ocular | **78,** 126 |
| **Other syndromes** | | | | | | | | |
| Autoimmune polyglandular syndrome type 3 (OMIM; ORPHA:227982) | No data; polygenic hereditary disease | 2 | 1/20 000 | M(1)/F(1), 6 | Presented at average age 6; Hashimoto’s thyroiditis, type I diabetes mellitus. | Presented at age 6 with patchy hair loss on scalp (patient 1); AA universalis with family history (patient 2); treated with local immunotherapy with squaric acid dibutylester for 3 months (n = 1) (partial resolution, lesion recurrence). |  | **48, 49**, 86, 87 |
| Deficient in anterior pituitary function-variable immunodeficiency (DAVID) syndrome (OMIM: #615577 ; ORPHA:293978) | *NFKB2*; autosomal dominant | 1 | <1/1 000 000 | M, 11 | Presented at 25 months; childhood onset of recurrent infections, hypogammaglobulinemia, ACTH insufficiency, and variable autoimmune features. | Presented at age 11; scalp; no treatment reported. |  | **65,** 105-107 |
| Louis-Bar Syndrome (OMIM: #208900; ORPHA:100) | *ATM*; autosomal recessive | 1 | 1-9/1 000 000 | F, 15 | No description reported. | No description reported. |  | **69,** 112 |

^a^ **Bold references:** original papers retrieved from the literature search; non-bold references: alternative sources used to provide complete information on each syndrome

^b^ Trisomy 21: Patients treated with methylprednisolone aceponate 0.1% cream and 5% minodoxil lotion (no resolution); tacrolimus ointment; oral steroid therapy; topical photochemotherapy with 8-methoxypsoralen, application of longwave ultraviolet light; tacrolimus 0.1% ointment with occlusion nightly for 6 months (complete resolution); 0.3% DNCB for 4 months (complete resolution); tofacitinib for 13 months (complete resolution); dithranol 0.2% in Vaseline with cystine tablets, cyclosporin A for 2-6 months (partial resolution); gluten free diet for 14 months (complete resolution)
